# Target Values and Daytime Variation of Bone Turnover Markers in Monitoring Osteoporosis Treatment After Fractures

**DOI:** 10.1002/jbm4.10633

**Published:** 2022-05-09

**Authors:** Tove T Borgen, Lene B Solberg, Trine Lauritzen, Ellen M Apalset, Åshild Bjørnerem, Erik F Eriksen

**Affiliations:** ^1^ Department of Rheumatology Vestre Viken Hospital Trust, Drammen Hospital Drammen Norway; ^2^ Division of Orthopedic Surgery Oslo University Hospital Oslo Norway; ^3^ Department of Laboratory Medicine Vestre Viken Hospital Trust, Drammen Hospital Drammen Norway; ^4^ Department of Clinical Medicine University of Oslo Oslo Norway; ^5^ Bergen Group of Epidemiology and Biomarkers in Rheumatic Disease, Department of Rheumatology Haukeland University Hospital Bergen Norway; ^6^ Department of Global Public Health and Primary Care University of Bergen Bergen Norway; ^7^ Department of Clinical Medicine UiT – The Arctic University of Norway Tromsø Norway; ^8^ Department of Obstetrics and Gynecology University Hospital of North Norway Tromsø Norway; ^9^ Norwegian Research Centre for Women's Health, Oslo University Hospital Oslo Norway; ^10^ Department of Endocrinology, Morbid Obesity and Preventive Medicine Oslo University Hospital Oslo Norway; ^11^ Department of Odontology University of Oslo Oslo Norway

**Keywords:** ANTIRESORPTIVES, BIOCHEMICAL MARKERS OF BONE TURNOVER, DXA, FRACTURE RISK ASSESSMENT, OSTEOPOROSIS

## Abstract

The serum bone turnover markers (BTM) procollagen type 1 N‐terminal propeptide (P1NP) and C‐terminal cross‐linking telopeptide of type 1 collagen (CTX) are recommended for monitoring adherence and response of antiresorptive drugs (ARD). BTM are elevated about 1 year after fracture and therefore BTM target values are most convenient in ARD treatment follow‐up of fracture patients. In this prospective cohort study, we explored the cut‐off values of P1NP and CTX showing the best discriminating ability with respect to adherence and treatment effects, reflected in bone mineral density (BMD) changes. Furthermore, we explored the ability of BTM to predict subsequent fractures and BTM variation during daytime in patients using ARD or not. After a fragility fracture, 228 consenting patients (82.2% women) were evaluated for ARD indication and followed for a mean of 4.6 years (SD 0.5 years). BMD was measured at baseline and after 2 years. Serum BTM were measured after 1 or 2 years. The largest area under the curve (AUC) for discrimination of patients taking ARD or not was shown for P1NP <30 μg/L and CTX <0.25 μg/L. AUC for discrimination of patients with >2% gain in BMD (lumbar spine and total hip) was largest at cut‐off values for P1NP <30 μg/L and CTX <0.25 μg/L. Higher P1NP was associated with increased fracture risk in patients using ARD (hazard ratio [HR]_logP1NP_ = 15.0; 95% confidence interval [CI] 2.7–83.3), *p* = 0.002. P1NP and CTX were stable during daytime, except in those patients not taking ARD, where CTX decreased by 21% per hour during daytime. In conclusion, P1NP <30 μg/L and CTX <0.25 μg/L yield the best discrimination between patients taking and not taking ARD and the best prediction of BMD gains after 2 years. Furthermore, higher P1NP is associated with increased fracture risk in patients on ARD. BTM can be measured at any time during the day in patients on ARD. © 2022 The Authors. *JBMR Plus* published by Wiley Periodicals LLC on behalf of American Society for Bone and Mineral Research.

## Graphical abstract

Daytime variation of (A) procollagen type 1 N‐terminal propeptide (P1NP) and (B) C‐terminal cross‐linking telopeptide of type 1 collagen (CTX) in patients using antiresorptive drugs (ARD) (yellow dots) and in patients not using ARD (blue triangles).

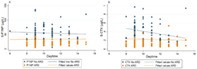

## 1 Introduction

Bone turnover markers (BTM) reflect bone remodeling activity and provide insight into how remodeling is affected by physiologic conditions, diseases, and drugs.^(^ ^1^ ^)^ In follow‐up of patients treated with antiresorptive drugs (ARD), BTM are important clinical tools for monitoring adherence and treatment effect.^(^ ^2^ ^)^ Two serum BTM are recommended to be measured at baseline and after 3 months of treatment with ARD: the bone formation marker procollagen type 1 N‐terminal propeptide (P1NP) and the bone resorption marker C‐terminal cross‐linking telopeptide of type 1 collagen (CTX).^(^ ^3^ ^)^ An expected response to antiresorptive treatment is defined as a reduction in BTM of more than the least significant change (LSC)^(^ ^4^ ^)^ or to a value below the mean levels of the premenopausal reference range.^(^ ^5^, ^6^, ^7^ ^)^ This corresponds to cut‐off values of P1NP <31.4 μg/L and CTX <0.30 μg/L, with the assays used in this study (Roche Cobas Elecsys).^(^ ^8^, ^9^ ^)^ After a fracture, however, the levels of BTM are elevated for up to 12 months.^(^ ^10^, ^11^ ^)^ This limits the use of BTM change in follow‐up when baseline values are assessed shortly after fragility fractures, a typical situation in a fracture liaison service (FLS) setting. Using a value below the mean of the premenopausal reference range as a treatment target in follow‐up is therefore more reasonable.^(^ ^5^, ^6^, ^7^ ^)^ Whether the same cut‐off values can be used for monitoring adherence and the treatment effects in patients with fragility fractures has, to our knowledge, not been studied. Another practical issue concerning BTM is that the blood samples should be collected in a fasting state in the morning before 10 a.m. This is especially important for measurement of CTX because it is influenced by food intake and exhibits significant diurnal variation.^(^ ^12^, ^13^ ^)^ Fasting morning sampling is not necessary for measurement of P1NP because of minimal diurnal variation.^(^ ^14^ ^)^ In real life, patients often do not attend fasting in the morning as requested, and to estimate variations in BTM during the day is therefore of interest for interpretation of BTM in a clinical setting. It is also of clinical interest whether BTM show a diurnal variation in patients using modern ARDs as alendronate, zoledronic acid, and denosumab.

The aims of this study were, therefore, in a cohort of patients with fragility fractures, to explore: (i) cut‐off values of P1NP and CTX that discriminate best patients' adherence to ARD; (ii) cut‐off values of P1NP and CTX that best predict treatment effects in terms of BMD change; (iii) whether P1NP and CTX predict fracture risk during follow‐up of patients using and not using ARD; (iv) variation in BTM by daytime in patients using or not using ARD.

## 2 Materials and Methods

### 2.1 Study subjects

The Norwegian Capture the Fracture Initiative (NoFRACT, NCT02536898) is a multicenter study at seven hospitals in Norway with 23,578 patients enrolled between May 2015 and January 2018.^(^ ^15^ ^)^ The main objective of NoFRACT was to investigate the effect of introducing a standardized FLS model of care on the rate of subsequent fractures. The intervention included identification of fracture cases, assessment, and treatment of osteoporosis in patients 50 years or older with recent fragility fractures. All types of fragility fractures were eligible, except fractures in fingers, toes, scull, and face. Anti‐osteoporotic treatment was recommended to patients with hip, vertebral, or two or more fragility fractures, to those with BMD *T*‐score ≤ −1.5 at either lumbar spine L_1_ to L_4_, total hip, or femoral neck, and/or 10‐year probability of major osteoporotic fracture (MOF; fracture of the hip, proximal humerus, wrist or clinical spine) assessed using the Fracture Risk Assessment Tool (FRAX score) ≥20%.

**Fig. 1:**
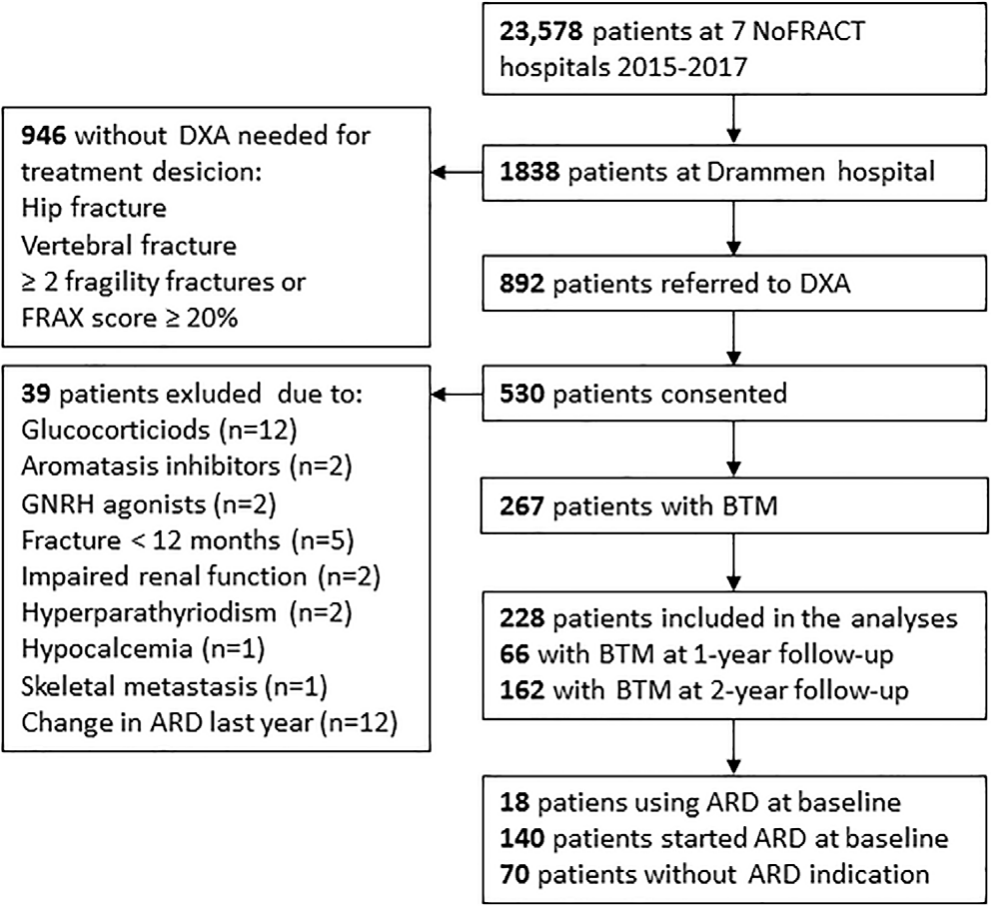
Flow chart of the study participants. NoFRACT = Norwegian Capture the Fracture Initiative; DXA = dual‐energy X‐ray absorptiometry; BTM = bone turnover markers; ARD = antiresorptive drugs; FRAX = 10‐year probability of major osteoporotic fracture (fracture of the hip, proximal humerus, wrist, or clinical spine) assessed using the fracture risk assessment tool; GNRH = gonadotropin‐releasing hormone agonist.

At Drammen Hospital, 1838 patients who were treated for a fragility between January 1, 2016, and December 31, 2017, were identified by FLS. Of those, 946 patients had either hip fracture, vertebral fracture, or FRAX score for MOF ≥20% and dual‐energy X‐ray absorptiometry (DXA) was not needed to evaluate treatment indication (Fig. 1). The remaining 892 patients were referred to DXA; of those, 530 patients consented to participate in a substudy (NCT02608801) of NoFRACT.^(^ ^16^ ^)^ In this article, we recruited 267 of those who all had BTM measured and were followed prospectively for mean 4.6 years. Baseline was the date when the patients attended DXA scan 2 to 12 weeks after the fracture and provided written consent to participate in the substudy. We excluded a total of 39 patients: 12 on treatment with glucocorticoids, 2 on aromatase inhibitors, 2 on gonadotropin‐releasing hormone agonists, 5 with a fracture during the last 12 months, 2 with impaired renal function (estimated glomerular filtration rate [eGFR] <45 nmol/min), 2 with hyperparathyroidism, 1 with hypocalcemia, 1 with cancer and skeletal metastasis, and 12 who had stopped or changed their ARD treatment the last 12 months. Hence, a total of 228 patients were included in the analyses, and 66 and 162 of them had BTM measured at 1‐year and 2‐year follow‐up. At baseline, 18 patients (9%) were already on ARD, and an additional 140 started ARD (alendronate [*n* = 121], denosumab [*n* = 15], and zoledronic acid [*n* = 22]); hence, 158 patients (69%) had prescribed ARD after baseline assessment, whereas 70 patients had no ARD prescribed because they did not have treatment indication (*T*‐score > −1.5 or FRAX‐score for MOF <20%). After 2‐year follow‐up, 145 of 158 patients were still on ARD (alendronate [*n* = 113], denosumab [*n* = 15], and zoledronic acid [*n* = 18]). Nine patients died during the total observation time of 4.6 years, but no one died during the first 2 years of follow‐up. All the participants had provided written informed consent. The Regional Committee for Medical and Health Research Ethics approved the study (REK 2014/2260).

### 2.2 Variables

Information about the use of and adherence to ARD was based on interview at baseline and follow‐up, and we further checked that the patients had received their drugs from the pharmacy using the prescription mediator database.

All had serum P1NP and CTX measured either at 1‐ or 2‐year follow‐up. BTM were not measured at baseline because all had recently sustained a fracture at that time and thus might exhibit falsely elevated values. All patients were recommended to fast for blood sampling between 8 a.m. and 10 a.m. Fasting status was not checked, and only 45.6% of the blood samples were collected before 10 a.m. The serum samples were collected and analyzed at once or stored at −80°C until analysis could be performed. Serum P1NP was measured using Elecsys Total P1NP immune assay on Cobas e 411 analyzer (Roche Diagnostics GmbH, Mannheim, Germany) with intra‐assay coefficient of variances (CVs) of 5.0% to 5.4% and interassay CVs of 2.0% to 4.4%.^(^ ^17^ ^)^ Serum CTX was measured using Elecsys β‐CrossLaps immune assay on Cobas Elecsys e 411 analyzer (Roche Diagnostics) with intra‐assay CVs of 3.7% to 4.1% and interassay CVs of <5.7%.^(^ ^9^ ^)^

At baseline and 2‐year follow‐up, height and weight were measured, and body mass index (BMI) was calculated as weight (kg) per square meter height. BMD was measured at lumbar spine (L_1_ to L_4_), total hip, and femoral neck at both hips using iDXA (GE Lunar, Pro, Madison, WI, USA). BMD measurements of the left hip were used in the statistical analyses. Lumbar vertebras with fracture were excluded from BMD assessment of the spine. The Third National Health and Nutrition Examination Survey reference data for female Whites aged 20 to 29 years was used for calculating BMD *T*‐scores of the hips.^(^ ^18^ ^)^ Daily phantom quality assurance (QA) of the iDXA was performed.

The patient's medical records were reviewed from baseline to September 2021, and each subsequent fractures were registered by type and date of fracture.

### 2.3 Statistical analyses

Continuous variables were presented as mean ± SD, and differences in means between groups were calculated using Student's *t* test. Categorical variables were reported as number (%), and groups were compared using Fisher's exact test. Continuous variables were checked for normality by inspection of histograms. The distribution of P1NP and CTX was left skewed; hence, these variables were reported as median with interquartile range and log‐transformed when used as continuous variables but not in the analyses when cut‐off values were tested.

Area under the receiver operating characteristic curve (AUC) analyses were performed to explore discrimination between groups of patients at cut‐off values for P1NP (<20, <25, <30, <35, <40 μg/L) and CTX (<0.20, <0.25, <0.30L, <0.35, <0.40 μg/L). The cut‐off value with the largest AUC was considered as the best to discriminate patients using versus not using ARD.

Similarly, the cut‐off value with the largest AUC was considered as the best to discriminate patients who gained BMD >2% versus those who did not gain BMD >2%. We decided a gain in BMD >2% was a clinical acceptable increase in BMD reflecting treatment effect of ARD, which also correspond to the surrogate threshold effect of ARD for total hip BMD on future fractures newly validated by Eastell and colleagues.^(^ ^19^ ^)^ The association between the change in BMD and BTM was tested using log‐transformed P1NP and CTX in linear regression analyses adjusted for sex, age, and hour of blood sampling. These results were further anti‐log transformed to enable interpretation.

The hazard ratio (HR) with 95% confidence interval (CI) for fracture by log‐transformed P1NP and log‐transformed CTX was calculated using Cox proportional hazards models adjusted for sex, age, BMI, hour of blood sampling, and total hip BMD in patients using and not using ARD.

Median P1NP and median CTX in blood samples obtained before and after 10 a.m. was calculated. The association between log‐transformed BTM and the hour of blood sampling was explored using linear regression models adjusted for age and sex in those using and not using ARD. The results were further anti‐log transformed to enable interpretation. The analyses were performed using Stata v15 (version 15, StataCorp, College Station, TX, USA).

## 3 Results

### 3.1 Characteristics of the fracture cohort according to treatment

The index fractures in 228 patients in the study were hip fractures (*n* = 23), forearm fractures (*n* = 101), proximal humerus fractures (*n* = 34), clinical vertebral fractures (*n* = 14), ankle fractures (*n* = 32), pelvic fractures (*n* = 7), and fractures at other sites (*n* = 18). After baseline assessment of fracture risk, 158 patients were prescribed ARD, and of these, 145 were still on ARD at 2‐year follow‐up (alendronate [*n* = 113], denosumab [*n* = 15], and zoledronic acid [*n* = 18]). The patients who continued to use ARD were older and had lower BMD at lumbar spine (L_1_ to L_4_), total hip, and femoral neck at baseline than those not on ARD (all *p* < 0.001) (Table 1). Patients using ARD had lower P1NP and CTX than untreated patients at both 1‐ and 2‐year follow‐up (Table 1; Fig. 1). After 2 years, there was a gain in BMD at all measured sites in the group using ARD, most at lumbar spine (6.3%). In the group not using ARD, there was a decline in BMD at all sites, the largest at femoral neck (−2.2%).

**Table 1:** Characteristics of Patients Using or Not Using Antiresorptive Drugs (ARD) at 2‐Year Follow‐Up

		ARD *n* = 145	No ARD *n* = 83
Baseline	Age, years (SD)	67.8 (7.9)^c^	62.8 (7.5)
	Women, *n* (%)	132 (90.4)^a^	69 (83.1)
	BMI, kg/m^2^ (SD)	25.2 (4.4)^b^	27.0 (4.0)
	BMD		
	L_1_ to L_4_, g/cm^2^ (SD)	0.954 (0.134)^c^	1.079 (0.133)
	*T*‐score L_1_ to L_4_ (SD)	−1.9 (1.1)^c^	−0.9 (1.0)
	Total hip, g/cm^2^ (SD)	0.778 (0.096)^c^	0.852 (0.089)
	*T*‐score total hip (SD)	−1.8 (0.8)^c^	−1.2 (0.7)
	Femoral neck, g/cm^2^(SD)	0.742 (0.086)^c^	0.813 (0.082)
	*T*‐score femoral neck (SD)	−2.1 (0.6)^c^	−1.6 (0.6)
	Lowest *T*‐score any site	−2.6 (0.6)^c^	−1.9 (0.5)
BTMs	P1NP year 1, μg/L (IQ)	20.0 (17.0, 23.0)^c^	38.5 (32.5, 63.0)
	P1NP year 2, μg/L (IQ)	20.0 (15.0, 25.0)^c^	53.0 (36.0, 68.0)
	P1NP year 1 + 2, μg/L (IQ)	20.0 (16.0, 24.0)^c^	53.0 (35.0, 68.0)
	CTX year 1, μg/L (IQ)	0.12 (0.09, 0.14)^c^	0.28 (0.12, 0.38)
	CTX year 2, μg/L (IQ)	0.12 (0.09, 0.16)^c^	0.37 (0.24, 0.53)
	CTX year 1 + 2, μg/L (IQ)	0.12 (0.08, 0.16)^c^	0.35 (0.24, 0.52)
Two‐year	BMD		
	L_1_ to L_4_, g/cm^2^ (SD)	1.014 (0.134)^b^	1.071 (0.131)
	L_1_ to L_4_ difference, g/cm^2^ (%)	0.060 (+6.3)^c^	−0.008 (− 0.7)
	Total hip, g/cm^2^ (SD)	0.805 (0.101)^b^	0.842 (0.077)
	Total hip difference, g/cm^2^ (%)	0.027 (+3.5)^c^	−0.010 (−1.2)
	Femoral neck, g/cm^2^(SD)	0.762 (0.097)	0.795 (0.074)
	Femoral neck difference (%)	0.020 (+2.7)	−0.018 (−2.2)
Observation period	Observation period, years (SD)	4.6 (0.5)	4.6 (0.5)
	Patients with fractures, *n* (%)	22 (15.1)	12 (14.5)
	Second fracture, *n* (%)	18 (12.4)	9 (10.8)
	Third fracture, *n* (%)	4 (2.8)	3 (3.6)
	Type of ARD used		
	Alendronate	113 (49.1)	‐
	Denosumab	15 (6.6)	‐
	Zoledronic acid	18 (7.9)	‐

BMI = body mass index; BMD = bone mineral density; P1NP = procollagen type I N‐terminal propeptide; s‐CTX = C‐terminal cross‐linking telopeptide of type I collagen; ARD = antiresorptive drugs; BTMs = bone turnover markers.

Values are mean ± standard deviation (SD), number (%) and median with interquartile range (IQ).

^a^ *p* <0.05.

^b^ *p* <0.01.

^c^ *p* <0.001.

During a mean observation period of 4.6 years, 22 patients (15.1%) using ARD experienced 26 subsequent fractures (1 hip, 4 forearm, 5 proximal humerus, 4 clinical vertebral, 4 ankle, and 8 other sites). In the group of patients not using ARD, 11 (14.5%) experienced 15 subsequent fractures (1 hip, 3 forearm, 1 proximal humerus, 1 clinical vertebral, 2 ankle, and 7 other sites). Nine patients died during the observation period: 3 in the alendronate group, 1 in the denosumab group, 3 in the zoledronic acid group, and 2 in those not using ARD.

### 3.2 Cut‐off values of P1NP and CTX for discrimination of patients using and not using ARD

AUC for discrimination of patients using versus not using ARD was largest using a cut‐off value for P1NP <30 μg/L (0.927) and a cut‐off value for CTX <0.25 μg/L (0.971) (Table 2; Fig. 3). These analyses included samples obtained before 10 a.m. (*n* = 104). We found the same results using P1NP samples from any time of the day (*n* = 228). For CTX, however, the largest AUC was found using cut‐off value <0.20 μg/L when using samples from any time of the day. The same cut‐off values were found if patients on denosumab and zoledronic acid were excluded.

**Table 2:** Area Under Curve (AUC) for Discriminating Patients on Antiresorptive Drugs at Different Cut‐Off Values of Procollagen Type 1N‐Terminal Propeptide (P1NP) and C‐Terminal Cross‐Linking Telopeptide of Type Collagen (CTX) (Time of Blood Sampling <10 a.m.)

P1NP	20 μg/L	25 μg/L	30 μg/L	35 μg/L	40 μg/L
	0.692	0.879	0.927	0.910	0.872

CTX	0.20 μg/L	0.25 μg/L	0.30 μg/L	0.35 μg/L	0.40 μg/L
	0.965	0.971	0.917	0.811	0.773

### 3.3 Cut‐off values of P1NP and CTX for treatment effect measured in BMD change

An increase in BMD in excess of 2% at lumbar spine, total hip, and femoral neck corresponded to an increase of more than 0.019 g/cm^2^, 0.016 g/cm^2^, and 0.015 g/cm^2^. The AUC for discrimination of patients with BMD gain 2% or more at lumbar spine, total hip, and femoral neck was largest using a cut‐off value for P1NP <30 μg/L (0.796, 0.701, and 0.626; Table 3). The largest AUC for BMD gain at the lumbar spine was a CTX cut‐off value <0.25 μg/L (0.851). The same results were found when patients on denosumab and zoledronic acid were excluded.

**Table 3:** Area Under Curve (AUC) for Achieving More Than 2% Increase in Bone Mineral Density (BMD) at Different Cut‐Off Values for Procollagen Type 1N‐Terminal Propeptide (P1NP) and C‐Terminal Cross‐Linking Telopeptide of Type I Collagen (CTX) in All Patients (Time of Blood Sampling for CTX < 10 a.m.)

>2% increase BMD		P1NP	20 μg/L	25 μg/L	30 μg/L	35 μg/L	40 μg/L
	L_1_ to L_4_		0.641	0.770	0.796	0.788	0.773
	Total hip		0.573	0.650	0.701	0.622	0.619
	Femoral neck		0.552	0.597	0.626	0.568	0.571

		CTX	0.20 μg/L	0.25 μg/L	0.30 μg/L	0.35 μg/L	0.40 μg/L
	L_1_ to L_4_		0.823	0.851	0.818	0.754	0.714
	Total hip		0.614	0.639	0.640	0.596	0.592
	Femoral neck		0.547	0.575	0.585	0.562	0.542

Lower log P1NP (β = −0.05, 95% CI −0.06, −0.03) (*p* < 0.001) and lower logCTX (β = −0.04, 95% CI −0.05, −0.03) (*p* < 0.001) were associated with larger gains in BMD after 2 years on ARD treatment (Fig. 4). After anti‐log transformation, lower P1NP (β = 0.89, 95% CI 0.87, 0.93) and lower CTX (β = 0.91, 95% CI 0.89, 0.93) were associated with larger gains in BMD after 2 years on ARD treatment.

### 3.4 Predictive value of P1NP and CTX for fractures during follow‐up

**Fig. 2:**
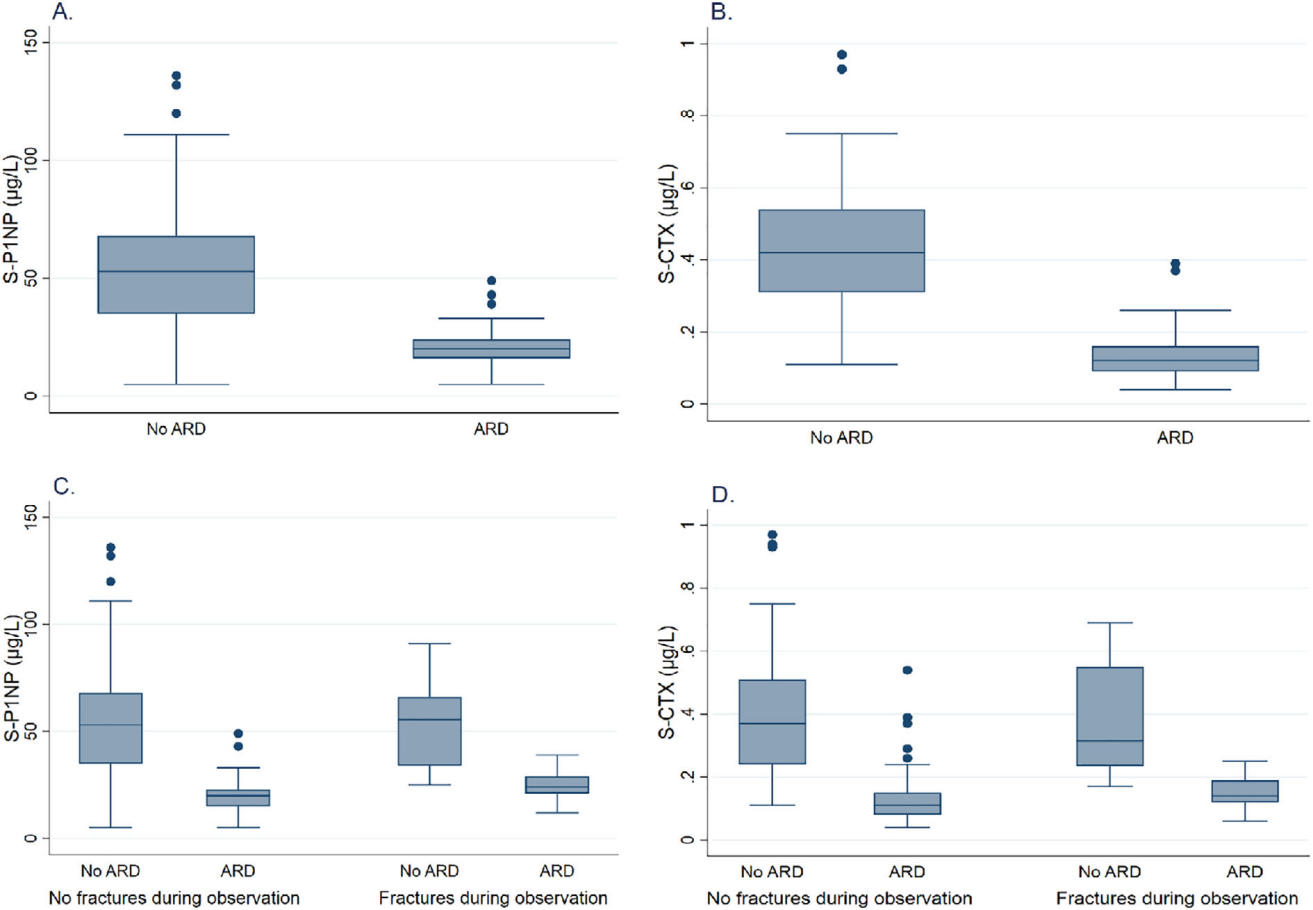
Box‐and‐whisker plots of (*A*) procollagen type 1 N‐terminal propeptide (P1NP) and (*B*) C‐terminal cross‐linking telopeptide of type 1 collagen (CTX) in the groups of patients not using and using antiresorptive drugs (ARD). (*C*) P1NP and (*D*) CTX in patients using and not using ARD, with and without fractures during follow‐up.

Neither median P1NP nor median CTX differed visibly in patients with and without subsequent fractures during the observation period (Fig. 2*C*, *D*). In patients using ARD, however, a higher log P1NP was associated with fracture risk during follow‐up with a HR of 15.0 (95% CI 2.71, 83.3) (*p* = 0.002) in models adjusted for age, sex, BMI, hour of blood sampling, and total hip BMD (Table 4). In patients using ARD, CTX was not associated with fracture risk. In patients not using ARD, P1NP and CTX were not associated with fracture risk.

**Table 4:** Hazard Ratio (HR) of Fracture During Follow‐Up With Log‐Transformed Procollagen Type 1N propeptide (logP1NP) and log transformed C‐terminal cross‐linking telopeptide of type 1 collagen (logCTX) in all patients, in the patients using and not using antiresorptive drugs (ARD)

			Univariable model		Multivariable model^a^ without BMD		Multivariable model^b^ with BMD	
			HR (95% CI)	*p*	HR (95% CI)	*p*	HR (95% CI)	*p*
All fractures	ARD	logP1NP	7.98 (2.12, 30.0)	0.002	6.87 (1.71, 27.6)	0.007	15.0 (2.71, 83.3)	0.002
		logCTX	2.30 (1.00, 5.31)	0.051	1.92 (0.79, 4.65)	0.150	2.21 (0.73, 6.66)	0.160

	No ARD	logP1NP	1.03 (0.37, 2.91)	0.952	1.06 (0.36, 3.17)	0.910	1.12 (0.37, 3.37)	0.847
		logCTX	0.82 (0.26, 2.57)	0.741	1.04 (0.31, 3.53)	0.944	1.05 (0.31, 3.53)	0.936

^a^ Including sex, age, body mass index, and time at day of blood sampling.

^b^ Including sex, age, body mass index, total hip bone mineral density, and hour of blood sampling.

**Fig. 3:**
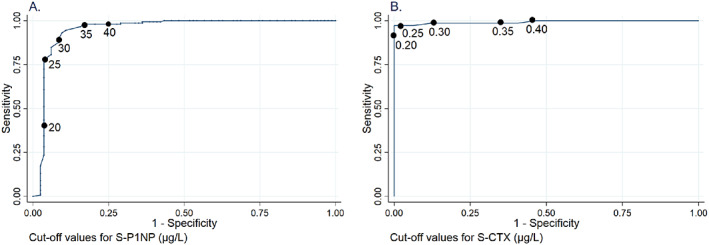
Area under the receiver operating curve for discrimination between patients using and not using antiresorptive drugs by (*A*) procollagen type 1 N‐terminal propeptide (P1NP) and (*B*) C‐terminal cross‐linking telopeptide of type 1 collagen (CTX) with different cut‐off values marked.

**Fig. 4:**
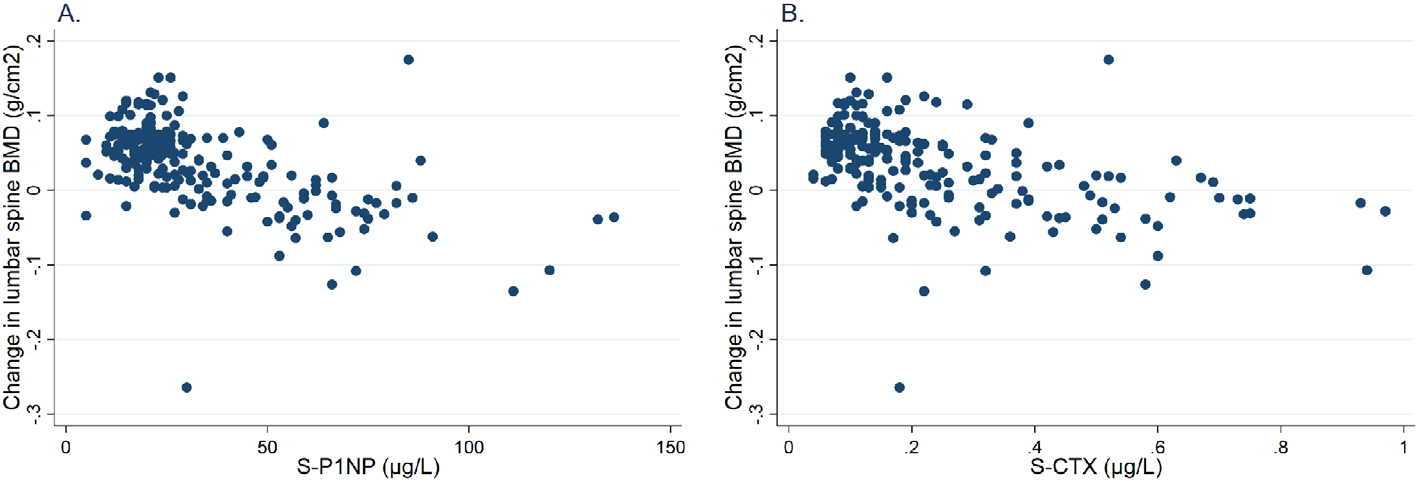
Scatter plots showing the association between change in lumbar spine bone mineral density (BMD) and (*A*) procollagen type 1 N‐terminal propeptide (P1NP) and (*B*) C‐terminal cross‐linking telopeptide of type 1 collagen (CTX) during 2‐year follow‐up. Time of blood sample for CTX < 10 a.m.

**Fig. 5:**
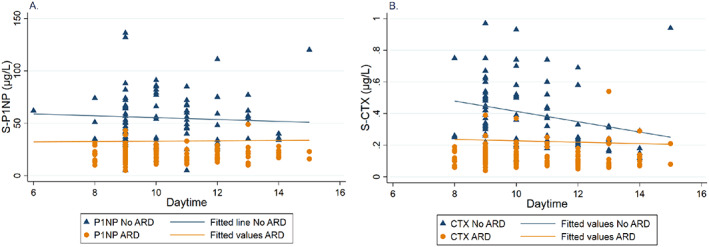
Scatter plots with fitted lines showing variation of (*A*) procollagen type 1 N‐terminal propeptide (P1NP) and (*B*) C‐terminal cross‐linking telopeptide of type 1 collagen (CTX) during the day in patients using antiresorptive drugs (ARD) (yellow dots) and in patients not using ARD (blue triangles).

### 3.5 Daytime variation in P1NP and CTX in the groups on ARD versus no ARD

In patients using ARD, there was no association between P1NP and CTX and hour of collection of blood sample (Table 5; Fig. 5). In patients not using ARD, P1NP remained stable during the day, but CTX decreased with 21% per hour (β = 0.79, 95% CI 0.68, 0.91) during the day.

**Table 5:** Median Serum Procollagen Type I N‐Terminal Propeptide (P1NP) and C‐Terminal Cross‐Linking Telopeptide of Type I Collagen (CTX) During the Day in the Group Using or Not Using Antiresorptive Drugs (ARD)

		Time of blood sampling		LogP1NP and logCTX difference per hour (95% CI)	Anti‐logP1NP and CTX difference per hour (95% CI)^a^
		<10 a.m. (*n* = 104)	>10 a.m. (*n* = 86)		
P1NP, μg/L	ARD	21.0	18.0	−0.01 (−0.04, 0.03)	0.98 (0.91, 1.07)
	No ARD	53.0	49.5	−0.01 (−0.08, 0.06)	0.98 (0.83, 1.15)

CTX, μg/L	ARD	0.12	0.11	0.00 (−0.04, 0.04)	1.00 (0.91, 1.10)
	No ARD	0.42	0.28	−0.10 (−0.17, −0.04)^b^	0.79 (0.68, 0.91)

^a^ Adjusted for age and sex.

^b^ *p* <0.01.

## 4 Discussion

In this cohort of FLS patients, who were assessed and offered ARD treatment after a fragility fracture, we explored several practical aspects pertaining to the use of BTM as tools for monitoring treatment. P1NP below a cut‐off level of 30 μg/L and CTX below 0.25 μg/L exhibited the best discrimination between patients using versus not using ARD. The same cut‐off values also yielded the best prediction of BMD gain after 2 years. In patients using ARD, a higher P1NP was associated with fractures during mean 4.6 years of observation time. Mean P1NP did not change according to the time for blood collection during the day among those using or not using ARD. CTX was also stable for samples taken during the day in patients using ARD but decreased during the day in patients not using ARD.

First, we corroborated the use of cut‐off values to assess treatment adherence and to discriminate patients using ARD from those not using ARD. An objection to this approach has been that not all patients are above this cut‐off level before starting treatment with ARD.^(^ ^20^ ^)^ We used area under the ROC curve analyses to explore the levels of P1NP and CTX with the highest sensitivity and specificity to capture adherence to ARD. The large AUCs, 0.927 for P1NP and 0.971 for CTX at cut‐off levels 30 and 0.25 μg/L, respectively, reveal that only a minority of the patients were false positive or false negative. This reflects the excellent ability of BTM to discriminate between adherent and nonadherent patients; also in a FLS setting, provided samples are collected later than 1 year after a fragility fracture.

The same cut‐off values for P1NP and CTX also yielded the best discrimination between patients with significant increases in BMD over 2 years on ARD treatment. The value of P1NP is somewhat lower than cut‐offs of 35 μg/L and 0.31 μg/L proposed by Eastell and Szulc, but variations according to different assays used must also be taken into account.^(^ ^1^ ^)^ The cut‐off value for CTX is in accordance with recommendations from other studies, with values below 0.25 to 0.28 μg/L.^(^ ^2^, ^4^, ^21^, ^22^ ^)^ These values also correspond to the geometric means of premenopausal women aged 35 years and older which is shown for P1NP and CTX of 31.4 μg/L and 0.25 μg/L, respectively.^(^ ^8^ ^)^ This has, to our knowledge, not been shown in an FLS cohort before.

The association between elevated levels of BTMs and increased fracture risk is a frequent subject of discussion. Although a clear association has not been demonstrated in many individual studies, some reviews and meta‐analyses infer a connection.^(^ ^23^ ^)^ In a meta‐analysis by Johansson and colleagues, a modest but significant association with fractures was found, but this association vanished when adjusting for BMD.^(^ ^24^ ^)^ Convincing evidence from the meta‐regression analyses of Bauer and colleagues demonstrated an association between BTM reductions and decrease in vertebral fractures in 28,000 patients treated with ARD.^(^ ^25^ ^)^ In our study, we demonstrated an association with P1NP and subsequent fractures in the patients who were using ARD but not in those not using ARD. These results are comparable to previous investigations on alendronate^(^ ^26^ ^)^ and zoledronic acid.^(^ ^27^, ^28^ ^)^ In our study, this association remained after adjustment for BMD, which suggests that P1NP may be an independent risk factor for fractures in patients treated with alendronate, zoledronic acid, and denosumab.

We explored BTM variations during daytime in patients on ARD versus untreated patients. In the group of untreated patients, a diurnal variation of CTX of 21% per hour was shown. This variation during daytime is well known,^(^ ^13^ ^)^ and Quist and colleagues demonstrated a variation in CXT of 15% per hour (80% change from 8 a.m. to 2 p.m.) in both premenopausal women, early and late postmenopausal women, and in men.^(^ ^29^ ^)^ Further, we showed in patients using ARD that P1NP and CTX showed no variations during daytime. This contradicts the findings of Quist and colleagues, who demonstrated that nasal salmon calcitonin was not able to break the circadian pattern of CTX. ^(^ ^29^ ^)^ Calcitonin was not one of the ARD used in our study, rather alendronate, zoledronic acid, and denosumab with more pronounced and prolonged antiresorptive effect, presumably strong enough to break the circadian pattern of BTM. This has, to our knowledge, not been described before. The diurnal variation has also been studied in patients using teriparatide, showing a larger amplitude in CTX than P1NP levels, and that the circadian variation BTM significantly differed according to the dosing time of the teriparatide treatment.^(^ ^30^ ^)^ None of the patients in our study used osteoanabolic treatment.

The faint BTM variation by daytime has important clinical implications as it permits reliable assessment of BTM during the whole day in patients on ARD, freeing up laboratory resources and being more convenient for the patients. In patients not using ARD, our data suggest that CTX values should be adjusted with 21% per hour taken after 10 a.m. The analyses were, however, not performed in the same patients during the day, but the effect of this possible confounder is probably small because of the large group size. We did not ensure that the patients were in a fasting state when the blood samples were collected, but feeding status is reported to have little impact on P1NP, whereas it exhibits a higher impact on CTX.^(^ ^31^ ^)^

This study has limitations. Patients in the group not using ARD were healthier and younger and had no indication for ARD. This was adjusted for in the multivariable models but not in the ROC analyses calculating cut‐off values. Fasting status was not ensured in the patients, and the BTM were not measured at the same year of follow‐up in all patients. We did not measure BTM in the same patients at different time points of the day. P1NP and CTX were measured using the automated electrochemiluminescence immunoassays by Roche. There are other commercial tests available, including radioimmunoassay and chemiluminescence for P1NP and enzyme‐linked immunosorbent and chemiluminescence assays for CTX.^(^ ^14^ ^)^ These can have other reference values; hence, the cut‐off values from our study is not necessarily generalizable. The strength of this study is the practical approach, demonstrating the usefulness of BTM in FLS and follow‐up after a fragility fracture. The compliant patients identified by BTM correspond to those who have an expected increase in BMD; hence, BTM can serve as a surrogate for BMD assessment in monitoring treatment effect.

In conclusion: (i) P1NP and CTX levels below 30 and 0.25 μg/L yield the best discrimination between patients using or not using ARD; (ii) P1NP and CTX levels below 30 and 0.25 μg/L yielded the best prediction for BMD gains after 2 years of ARD treatment; (iii) P1NP can predict fractures in patients on ARD; and (iv) assessment of BTM can be extended to the whole day in patients on ARD. Thus, BTM constitute a valuable supplement to DXA assessment of effects of osteoporosis treatment and might replace DXA in some instances. However, DXA is still needed for decisions with respect to diagnosis, assessment of treatment goals, and treatment pauses.

## Disclosures

TTB has received speaker fees from UCB and Amgen and has served on the advisory board for UCB. EFE has received speaker fees from Novartis, Eli Lilly, Amgen, MSD, EffRx, IDS, UCB, Roche, and Shire. All other authors state that they have no conflicts of interest.

## Author contributions

**Tove T Borgen:** Conceptualization; data curation; formal analysis; investigation; methodology; project administration; visualization; writing – original draft. **Lene B Solberg:** Conceptualization; funding acquisition; project administration; supervision; writing – review and editing. **Trine Lauritzen:** Methodology; validation; writing – review and editing. **Ellen M Apalset:** Conceptualization; methodology; writing – review and editing. **shild Bjrnerem:** Conceptualization; data curation; funding acquisition; methodology; project administration; supervision; writing – review and editing. **Erik F Eriksen:** Conceptualization; methodology; supervision; writing – review and editing.

## Authors' roles

TTB, ÅB, and EFE led the design of this substudy. TTB led the patient involvement and data collection. All authors contributed to methodological decisions, data interpretation, conclusions, and dissemination. TTB performed the statistical analysis. TTB and EFE drafted the initial manuscript and are responsible for the data integrity. All authors contributed to editing of the manuscript and agreed on the final manuscript. ÅB is the chief investigator, leading protocol development, approvals, and dissemination.

5

### Peer review

The peer review history for this article is available at https://publons.com/publon/10.1002/jbm4.10633.
